# The efficacy and safety of acupuncture for patients with post-percutaneous coronary intervention depression: A protocol for systematic review and meta analysis

**DOI:** 10.1097/MD.0000000000023510

**Published:** 2020-12-11

**Authors:** Yong Liu, Disha Dai, Kailin Huang, Rui Zhuang, Liyong Ma, Birong Liu, Yi Pan, Lijing Zhang

**Affiliations:** a Department of Cardiology, Dongzhimen Hospital, Beijing University of Chinese Medicine, Beijing; b Department of Cardiology, People's Hospital of Wuhan University, Wuhan, Hubei; c Department of Cardiology, Dongfang Hospital, Beijing University of Chinese Medicine, Beijing, China.

**Keywords:** acupuncture, depression, percutaneous coronary intervention, protocol, systematic review

## Abstract

**Background:**

Percutaneous coronary intervention (PCI) has been increasingly applied as an effective revascularization strategy in patients with coronary artery disease (CAD). However, recent studies had indicated a higher incidence of depression on post-PCI patients. Acupuncture therapy is effective for depression. However, the treatment effect of depression on post-PCI patients is still not clear. Therefore, this systematic review and meta-analysis protocol is planned to evaluate the efficacy and safety of acupuncture for depression in post-PCI patients.

**Methods:**

Six English databases (PubMed, Web of science, Medline, EMBASE, Springer Cochrane Library and WHO International Clinical Trials Registry Platform) and 4 Chinese databases (Wan fang Database, Chinese Scientific Journal Database, China National Knowledge Infrastructure Database (CNKI) and Chinese Biomedical Literature Database) will be searched normatively according to the rule of each database from the inception to August 1, 2020. Two reviewers will independently conduct article selection, data collection, and risk of bias evaluation. Any disagreement will be resolved by discussion with the third reviewer. Either the fixed-effects or random-effects model will be used for data synthesis based on the heterogeneity test. The change in the scores on the Hamilton depression scale (HAMD) and the Self-rating depression scale (SDS) will be used as the main outcome measure. All-cause mortality, cardiac mortality, Major Adverse Cardiovascular Events (MACEs), rehospitalisation rate and Quality of Life Scale (SF-36) as the secondary outcome. Treatment Emergent Symptom Scale (TESS), General physical examination (temperature, pulse, respiration, blood pressure), Routine examination of blood, urine and stool, Electrocardiogram, Liver and kidney function examination as the security indexs. RevMan5.3.5 will be used for meta-analysis.

**Results:**

This study will provide high-quality evidence to assess the efficacy and safety of acupuncture for depression in post-PCI patients.

**Conclusion:**

This systematic review will explore whether acupuncture is an effective and safe intervention for depression in post-PCI patients.

## 1 Introduction

Cardiovascular disease is the infamous global health care burden and the world's biggest killer (WHO, 2011), while Coronary artery disease (CAD) ranked top on the list.^[1–3]^ With the advantage of miniature damage and optimal effectiveness, percutaneous coronary intervention has become the most effectively therapeutic and important way to restore blood flow in CAD patients.^[4,5]^ However, recent studies had indicated a higher incidence of depression on post-PCI patients.^[6–8]^ This will not only devastate the mental health of patients, it also cast shadows on the prognosis of post-PCI maneuvers.^[9]^ A 10-year long prospective research which included 1411 post-PCI patients indicated a 24.8% prevalence rate of depression, an increase of 77% was observed on all-cause mortality when compared to patients without depressive symptoms.^[9]^ The results of a meta-analysis show that perioperative diagnosis of depression was associated with a 2.1-fold increase in MACEs incidence in CAD patients after PCI.^[10]^

Post-PCI depression consists mainly of psychotherapy and antidepressant medications,^[11]^ but both exhibit certain extent of deficiencies, therefore failing to reach an ideal state of recovery. Antidepressant treatment is often accompanied by various adverse effects,^[12]^ such as sexual dysfunction, weight changes and gastrointestinal symptoms. Most importantly, when combined with medications in secondary preventions of coronary artery disease, drug interactions will be triggered. For example, various recent guidelines across the world had place Selective Serotonin reuptake inhibitors (SSRIs) as the first line anti-depressant^[13]^ while post-PCI patients often has to correspondingly undergo a guideline recommended 1-year regular intake of anticoagulants and dual antiplatelet therapy.^[14]^ Recent studies had correlated the combined medications of SSRIs, anticoagulants and dual antiplatelet with a higher risk of hemorrhage.^[15,16]^ Psychotherapy on the other hand, despite exhibiting no obvious adverse effects, the high treatment cost and acute shortage of psychotherapists had substantially limited its clinical application scale.^[17,18]^ Therefore, recruiting a safe, effective, and economic complementary and alternate therapy, while enhancing the secondary preventions of coronary artery disease is of utmost importance.

Acupuncture is the world renowned ancient Chinese therapy which had long served China's medical system for more than 3000 years. The theories employed in acupunctures are derived from the concept of holism, viscera, and meridians in Traditional Chinese Medicine (TCM). Therapeutic effects are achieved by regulating qi, blood, Yin and Yang of the human body.^[7]^ Previous meta-analysis proved acupuncture had satisfactory results on both efficacy and safety in the treatment of depression.^[13]^ Results from animal testing showed acupuncture could serve the role of an anti-depressant, through it multi-targets stimulations, it might be closely related to the regulations of monoamine neurotransmitters, inflammatory factors and neuroendocrine factors.^[19]^ In addition, meta-analysis showed that acupuncture combined with conventional medications could relieve the symptoms and reduce the duration of angina pectoris while enhancing recovery.^[20]^ However, the efficacy and safety of acupuncture for post-PCI depression treatment remains unclear, this study is the attempt to evaluate the enquiry through a systematic review and meta-analysis.

## 2 Methods

### 2.1 Study registration

This review protocol is registered in the PROSPERO International Prospective Registerof systematic reviews, registration number CRD42020207813. Available from: https://www.crd.york.ac.uk/prospero/display_record.php?RecordID=207813, and has been reported following the Preferred Reporting Items for Systematic Reviews and Meta-analyses guidelines.^[21]^

### 2.2 Inclusion criteria for study selection

#### 2.2.1 Types of studies

All relevant randomized controlled trials (RCTs) in English and Chinese will be included. While Non-RCTs, quasi-RCTs, cohort studies, reviews, case reports, experimental studies, expert experience, the data of the included study is missing or incomplete, and duplicate publications will be excluded to ensure the quality of this systematic review.

#### 2.2.2 Types of participants

Participants of different age groups with depression following PCI could be included in the study, regardless of nationality, race, gender, occupation, and educational background. While the cause of depression is not limited, experimental objects which included patients with schizophrenia would be excluded.

#### 2.2.3 Types of interventions

This study focuses on the RCTs of depression under the treatment of acupuncture. We will accept all types of acupuncture interventions without any restrictions of their acupuncture point, duration, and frequency. The treatment group should be treated by acupuncture combining or not combining with western medicines. The results are anticipated to aid clinicians. All trials with an assessment of the treatment mentioned above will be included, while studies of control group could only use western medicines as the sole treatment.

#### 2.2.4 Types of outcome measures

##### 2.2.4.1 Primary outcomes

The primary outcomes are the Hamilton depression scale (HAMD) and the Self-rating depression scale (SDS).

##### 2.2.4.2 Secondary outcomes

The secondary outcomes of this review mainly include the following aspects:

1. All-cause mortality, cardiac mortality.
2. Major Adverse Cardiovascular Events (MACEs).
3. Rehospitalization rate.
4. Quality of Life Scale (SF-36).

##### 2.2.4.3 Security index

1. Treatment Emergent Symptom Scale (TESS).
2. General physical examination (temperature, pulse, respiration, blood pressure).
3. Routine examination of blood, urine and stool.
4. Electrocardiogram.
5. Liver and kidney function examination.

### 2.3 Data sources

Six English databases (PubMed, Web of science, Medline, EMBASE, Springer Cochrane Library, and WHO International Clinical Trials Registry Platform) and 4 Chinese databases (Wan fang Database, Chinese Scientific Journal Database, CNKI, and Chinese Biomedical Literature Database) will be searched normatively in accordance with the rule of each database from the inception to August 1, 2020.

### 2.4 Searching strategy

Search strategy will be built in accordance with the guidelines from the Cochrane handbook. The Search strategy for PubMed is shown in Table 1, which included all search terms, and similar strategies will be built and applied for other electronic databases.

**Table 1:** The search strategy for PubMed.

Number	Search terms
#1	(Depression [Mesh]) OR (((((((((Depressions [Title/Abstract]) OR (Depressive Symptoms [Title/Abstract])) OR (Depressive Symptom [Title/Abstract])) OR (Symptom, Depressive [Title/Abstract])) OR (Symptoms, Depressive [Title/Abstract])) OR (Emotional Depression [Title/Abstract])) OR (Depression, Emotional [Title/Abstract])) OR (Depressions, Emotional [Title/Abstract])) OR (Emotional Depressions [Title/Abstract]))
#2	(Percutaneous Coronary Intervention[Mesh]) OR (((((((((((Coronary Intervention, Percutaneous [Title/Abstract]) OR (Coronary Interventions, Percutaneous [Title/Abstract])) OR (Intervention, Percutaneous Coronary [Title/Abstract])) OR (Interventions, Percutaneous Coronary [T itle/Abstract])) OR (Percutaneous Coronary Interventions [Title/Abstract])) OR (Percutaneous Coronary Revascularization [Title/Abstract])) OR (Coronary Revascularization, Percutaneous [Title/Abstract])) OR (Coronary Revascularizations, Percutaneous [Title/Abstract])) OR (Percutaneous Coronary Revascularizations [Title/Abstract])) OR (Revascularization, Percutaneous Coronary [Title/Abstract])) OR (Revascularizations, Percutaneous Coronary [Title/Abstract]))
#3	(Acupuncture Points[Mesh]) OR (((((Acupuncture Point[Title/Abstract]) OR (Point, Acupuncture [Title/Abstract])) OR (Points, Acupuncture[Title/Abstract])) OR (Acupoints [Title/Abstract])) OR (Acupoint [Title/Abstract]))
#4	(RCT [Title/Abstract]) OR (randomized controlled trial [Title/Abstract])
#5	(efficacy [Title/Abstract]) OR (safety [Title/Abstract])
#6	#1 and #2 and #3 and #4 and #5

This search strategy will be modified as required for other electronic databases.

### 2.5 Data collection and analysis

#### 2.5.1 Selection of studies

**Figure 1:**
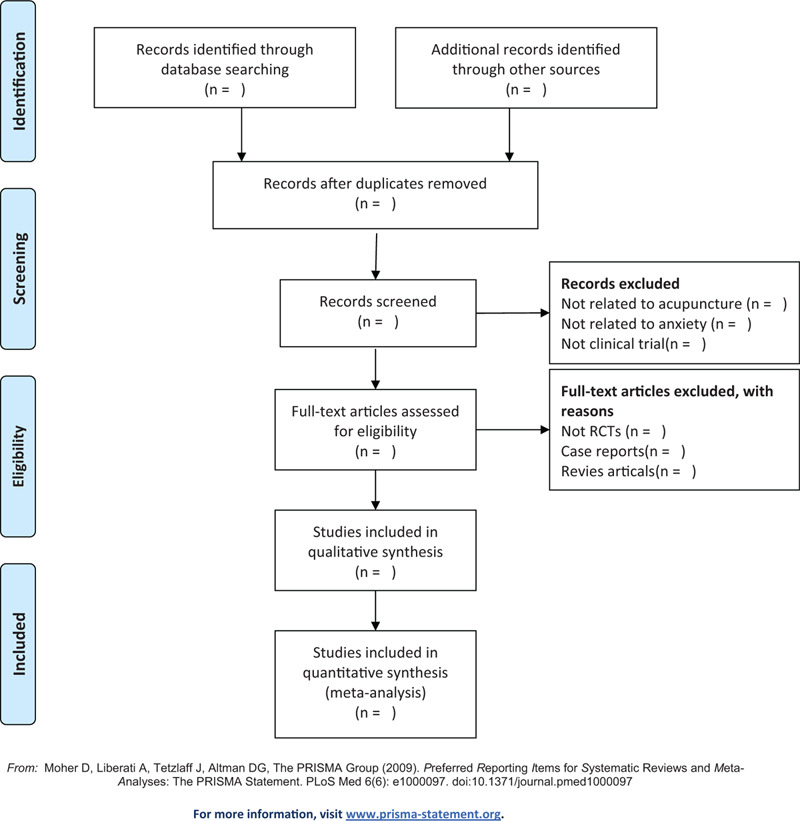
The PRISMA flow chart of the selection process.

The basic process of including literature will be pursued in reference to the Cochrane Collaboration System Evaluator's Manual (5.1.0). Relevant literatures will be obtained from specified databases, later imported into an Endnote X9 created database. Duplicate documents will be screened out through this process. Independent screening of titles, abstracts, and keywords of all retrieved records will be performed by 2 researchers. The name of the study, author, publishing year, country, database, and justification of the study meeting eligibility criteria to be therefore included in the review will be documented within an excel spreadsheet. Reasons of inclusion and exclusion (PICOS) are disclosed in a spreadsheet during abstract screening and full-text evaluation. A third researcher will be required on making the final decision to resolve any disagreement among the 2 researchers on literatures. The screening flow diagrams of this study will be shown in Figure 1.

#### 2.5.2 Data extraction and management

Two independent reviewers will extract the data of interest from the eligible study and fill in the data collection sheet. If consensus on data extraction is failed to reach by discussion, the decision will be made by the third reviewer. Microsoft Excel 2013 will be used for data and information management. We will extract the following data:

1. The basic characteristics of RCT: title, 1st author, publishing year, country, and the journal.
2. Participants’ characteristics: average age, gender, sample size, inclusion and exclusion criteria, baseline situation, type, and criteria for the classification of depression.
3. Interventions: treatment duration, study design, randomization, allocation concealment, and blinding methods.
4. Comparators: western medicines.
5. Outcomes: measures, primary and secondary outcomes, security indexes, and follow up.

#### 2.5.3 Assessment of risk of bias

Cochrane bias risk tool (RevMan 5.3.5) will be employed to evaluate the risk of bias, while the following 6 domains will be assessed: random sequence generation, allocation concealment, blinding, incomplete outcome data, selective reporting, and other bias. Each potential trial of bias will be graded as high, low, and unclear. When the 2 independent reviewers failed to reach a consensus on the risk of bias assessment by negotiation, a third reviewer will make a final decision.

#### 2.5.4 Measures of treatment effect

Mean differences (MD) or standard mean difference (SMD) with 95% CIs will be used as continuous data, and the dichotomous outcomes will be estimated by the risk ratio (RR) with 95% confidence intervals (CIs).

#### 2.5.5 Unit of analysis issues

Only the 1st experimental period data of crossover trials will be extracted in order to minimize carryover effects. For trials regarding multiple interventions, all relevant experimental groups and control groups within the trial will be combined into a single group to avoid unit-of-analysis error.

#### 2.5.6 Management of missing data

For missing data, we will first try to contact the original author. The research would be excluded from the study if the data failed to be provided on request.

#### 2.5.7 Assessment of heterogeneity

Visual inspection of the forest plots and standard *χ*2 test and *I*^2^ test will be employed to assess heterogeneity. When *P* > .1, *I*^2^ < 50%, it will be considered as no significant heterogeneity between the trials, and the fixed effect model will be applied for statistics, otherwise, the random effect model will be chosen. When heterogeneity occurs, sensitivity analysis or meta regression will be performed to assess the source of heterogeneity.

#### 2.5.8 Assessment of reporting biases

If 10 or more studies are included in the meta-analysis, funnel plots and Egger test will be used to evaluate the reporting bias. The trim and fill method will be applied to identify and correct asymmetric funnel arising from publication bias, if appropriate.^[22]^

#### 2.5.9 Data synthesis

Data analysis and synthesis will be performed using RevMan version 5.3 software provided by the Cochrane Collaboration. The software will be used to obtain forest plots and test the heterogeneity between the included studies. Risk ratio (RR) with 95% CIs will be used for dichotomous data, while the continuous data will be analyzed by mean difference (MD) or standard MD (SMD) with 95% CIs. Heterogeneity will be assessed by visual inspection of the forest plots and detected by standard *χ*2 test and *I*^2^ test. When *P* > .1, *I*^2^ < 50%, it will be considered as no significant heterogeneity between the trials, and the fixed effect model will be applied for statistics, otherwise, the random effect model will be chosen. When heterogeneity occurs, sensitivity analysis or meta regression will be performed to assess the source of heterogeneity.

#### 2.5.10 Subgroup analysis

When heterogeneity is detected, subgroup analysis will be used (e.g., different types of western medicines therapies, patient conditions, research quality, publication age, and participation population) to spot the source of heterogeneity.

#### 2.5.11 Sensitivity analysis

In trials with sufficient data, sensitivity analyses will be taken to test the robustness and reliability of the results. Our sensitivity analysis will be based on heterogeneity, sensitivity analysis may be performed, and certain low-quality or unblinded studies would be excluded when heterogeneity occurs.

## 3 Discussion

High incidence of post-PCI depression would not only corrupt the physical and mental health of patients, but also threaten the prognosis of post-PCI.^[23,24]^ Pharmacotherapy and psychotherapy are the general treatments for post-PCI depression, but neither is ideal. Pharmacotherapy retained potential adverse effects such as sexual dysfunction, weight fluctuation, and gastrointestinal symptoms. Drug interactions should also be brought to attention when the application of antidepressants is combined with cardiovascular medicine.^[25–30]^ Psychotherapy is difficult to popularize because of the high cost of treatment and professional requirements demanded on healthcare personnel. Acupuncture on the other hands, has a long history of usage throughout China, Japan, and Korea. Several systematic reviews and meta-analysis have proved the acupuncture safe and effective on the treatment of depression and CAD.^[16,20,31,32]^ However, the safety and efficacy of acupuncture treatment on post-PCI depression remains unclear. This systematic review and meta-analysis will provide a convincing conclusion to justify the efficacy and safety of acupuncture for post-PCI patients. The conclusion drawn from this review is anticipated to assist clinicians on the treatments of post-PCI depression and benefits corresponding patients. Clues and result derived from this study intends to encourage researchers to conduct further research on the subject.

## Author contributions

YL, DSD, KLH, YP, and LJZ conceived and designed the protocol, and YL drafted the protocol manuscript. YL developed the search strategy, with input from BRL and RZ. LYM and RZ planned the data extraction. YL, DSD, and BRL planned the quality appraisal of all included studies. YL, KLH, YP, RZ, BRL, LYM, and LJZ critically revised the manuscript for methodological and intellectual content. All authors approved the final version.

**Conceptualization:** Yong Liu, Disha Dai, Kailin Huang, Birong Liu, Lijing Zhang.

**Data curation:** Yong Liu, Kailin Huang, Birong Liu.

**Formal analysis:** Rui Zhuang, Birong Liu, Yi Pan.

**Project administration:** Yong Liu, Liyong Ma, Yi Pan.

**Supervision:** Yong Liu, Rui Zhuang, Liyong Ma, Birong Liu.

**Writing – original draft:** Yong Liu, Disha Dai, Yi Pan.

**Writing – review & editing:** Yong Liu, Lijing Zhang.
